# The Public and the General Practitioner

**Published:** 1916-07-15

**Authors:** 


### THE PUBLIC AND THE GENERAL PRACTITIONER.

Of the urgency of personal calls for the doctor most households have had experience, and just now a national demand of a similar order is prominent. This latter is gradually being satisfied and its satisfaction must inevitably make some difficulty in meeting the more personal need. Not even the medical practitioner can be in two places at once, and when he is sent to the Army he is necessarily beyond the reach of the urgent message. Unless the medical profession is greatly in excess of civil requirements—and few will support such a proposition—the present position must mean that the private practitioner is much less available than he was prior to the war. There is certainly no sensible diminution of the amount of illness, for the sending of two or three millions of strong and healthy men out of the country can make little difference in this respect, and there are factors in the situation which tell in the opposite direction. On the other hand, the number of doctors at hand for the wants of the civil population has been reduced by some 30 per cent. In some measure the discrepancy has been met by lowering the activity of certain organised medical activities, such, for example, as those concerned with public health, with the inspection of school children, and with hospital opportunities. But even when full allowance is made for all these, it must still be true that the public cannot find the practitioner with the readiness to which it has long been accustomed. And readiness is one of the needs of the situation. Skill and competence, of course, are desired, but also promptness to meet an emergency. Hence it may well be that the existing situation will make conspicuous the interest which the public has in an adequate supply of doctors if accidents and other acute contingencies are to receive ready attention. This, in other words, means an adequate supply of doctors engaged in general practice, for it is by these alone that the daily needs of the situation can be quickly and efficiently satisfied.

But, as in other callings, general practice will certainly lose recruits if its outlook and prospects are rendered unattractive. Now there are certain existing influences which tend to this result. Some of these cannot be avoided, as, for example, the removal of many cases of the infectious fevers from the care of the private practitioner to isolation hospitals. But within recent years there have been advanced a number of other proposals which if adopted will mean an even more serious invasion of the opportunities of the general practitioner. Schemes connected with the organised care of maternity, of school children, of infants, and for the promotion of child welfare, are numerous, and their promoters are largely disposed to rely for medical aid on appointed whole-time officers. We say nothing in opposition to these schemes, but their suggested medical organisation demands careful consideration from the point of view with which we are here concerned. It is in the public interest that they shall so far as possible be worked by doctors engaged in general practice in the neighbourhood of their activities, as by this means alone can the attractions of general practice be maintained at such a level as will lead well-qualified men to undertake it. Reduce these attractions below a certain point and both the number of doctors at the ready summons of the public and the promptness of medical aid will certainly decline.

